# Climate change and plant genomic plasticity

**DOI:** 10.1007/s00122-025-05010-x

**Published:** 2025-08-27

**Authors:** Carlo M. Pozzi, Angelo Gaiti, Alberto Spada

**Affiliations:** 1 Dipartimento Di Scienze Agrarie Ed Ambientali, Università Di Milano, Via Celoria 2, 20133 Milan, Italy; 2 Dipartimento Di Scienze Per Gli Alimenti, La Nutrizione E L’Ambiente, Università Di Milano, Via Celoria 2, 20133 Milan, Italy

## Abstract

**Key message:**

Genome adaptation, driven by mutations, transposable elements, and structural variations, relies on plasticity and instability. This allows populations to evolve, enhance fitness, and adapt to challenges like climate change.

**Abstract:**

Genomes adapt via mutations, transposable elements, DNA structural changes, and epigenetics. Genome plasticity enhances fitness by providing the genetic variation necessary for organisms to adapt their traits and survive, which is especially critical during rapid climate shifts. This plasticity often stems from genome instability, which facilitates significant genomic alterations like duplications or deletions. While potentially harmful initially, these changes increase genetic diversity, aiding adaptation. Major genome reorganizations arise from polyploidization and horizontal gene transfer, both linked to instability. Plasticity and restructuring can modify Quantitative Trait Loci (QTLs), contributing to adaptation. Tools like landscape genomics identify climate-selected regions, resurrection ecology reveals past adaptive responses, and pangenome analysis examines a species’ complete gene set. Signatures of past selection include reduced diversity and allele frequency shifts. Gene expression plasticity allows environmental adaptation without genetic change through mechanisms like alternative splicing, tailoring protein function. Co-opted transposable elements also generate genetic and regulatory diversity, contributing to genome evolution. This review consolidates these findings, repositioning genome instability not as a mere source of random error but as a fundamental evolutionary engine that provides the rapid adaptive potential required for plant survival in the face of accelerating climate change.

**Supplementary Information:**

The online version contains supplementary material available at 10.1007/s00122-025-05010-x.

## Role of genome plasticity in plant adaptation to climatic changes

**Fig. 1:**
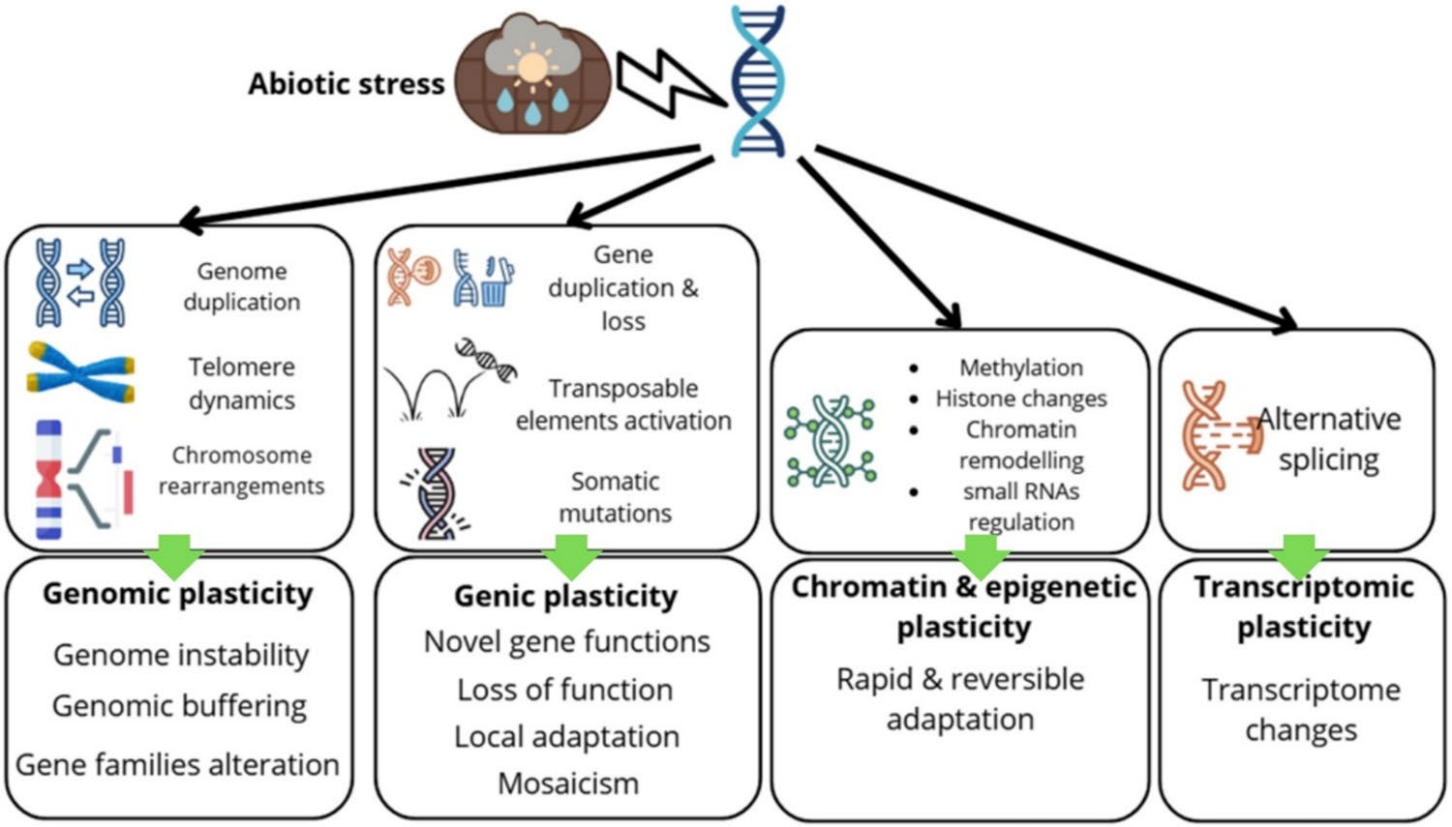
Plant genomic plasticity mechanisms under climate change-induced abiotic stress involve diverse genomic, genic, chromatin-epigenetic, and transcriptomic adaptations that collectively contribute to genome instability, novel gene functions, reversible epigenetic modifications, and transcriptomic changes, enhancing plant resilience and adaptation. The green arrows illustrate how each set of molecular mechanisms (top)—such as genome duplication or methylation—gives rise to a specific category of plasticity and its functional consequences (bottom). Genic plasticity refers to stress-induced changes occurring at the level of individual genes, leading to new genetic variations. Chromatin and epigenetic plasticity involves modifications to the chromatin structure that regulate gene accessibility and expression without altering the underlying DNA sequence. These responses are often rapid and reversible, allowing plants to adapt flexibly to environmental stress. Transcriptomic plasticity refers to changes at the RNA level that allow for flexible adaptation to the environment without any genetic change

Abiotic stressors, such as water stress, temperature changes, nutrient imbalances, and radiation, harm plant growth and development. These factors cause genotoxic and oxidative stress, damaging DNA and cellular functions. Climate change intensifies stress, leading to genomic instability, mutations, or cell death, reducing plant health and crop yield (Bartas 2024; Monroe 2023)(Fig. 1). Plant species respond to climate change through shifts to higher latitudes, altitudes, and earlier flowering (Anderson and Song 2020). *Mimulus laciniatus* populations revealed that drought-adapted descendants exhibited earlier seedling germination than their pre-drought ancestors, demonstrating an adaptation to stress (Dickman et al. 2019).

A key possible consequence of species spreading in response to climate change is the erosion of genetic diversity and fitness, which leads to a significant shift in the population’s genetic makeup (Exposito-Alonso et al. 2019). Given rapid environmental changes, understanding how plants cope with current climate variation is crucial. A key question is whether existing genetic plasticity can adequately facilitate necessary adaptive responses, considering the contribution of genotype-by-environment interaction to phenotypic variation. Phenotypic plasticity, a genotype’s ability to produce different phenotypes in varying environments, offers a short-term adaptive strategy. Environmental changes force this plasticity as an alternative to migration, allowing plants to buffer against immediate climate challenges without requiring genetic changes. However, plasticity may not suffice for intensifying environmental pressures, necessitating stress-specific genetic adaptation (Sommer 2020). Distinguishing the roles of phenotypic plasticity and genetic contributions is challenging, leading to the assumption that phenotypic plasticity is the more immediate plant response to climate stresses. Genomic plasticity is the inherent capacity of an organism’s genome to undergo structural and functional changes in response to varying environmental conditions. On the other hand, phenotypic plasticity is the ability of a single genotype to produce different phenotypes, for example under abiotic stresses. Genomic plasticity reflects in genomic responses, i.e., alterations to the structure, organization, or sequence of an organism’s DNA, often triggered by environmental stress.

Plant adaptation involves immediate gene activity changes, enabling phenotypic plasticity, and long-term genetic divergence in genes supporting this plasticity. These genetic differences can lead to distinct plant sub-populations better suited to local environments (Napier et al. 2023). Identifying the genes responsible for adaptive phenotypic variations is a goal in evolutionary biology. In Arabidopsis, genes with variable responses to drought and cold stress have a significantpolymorphism in their promoter regions (Lasky et al. 2014), like in the *CBF* locus, regulating cold-responsive pathways and freezing tolerance. Here, specific alleles enhancing freezing tolerance in cold climates might be detrimental in warmer regions, leading to their loss or modification (Gehan et al. 2015). This exemplifies adaptation through genetic compensation, a mechanism that allows organisms to maintain fitness despite genomic disruptions (Monroe 2023; Novillo et al. 2004). However, it also has implications for predicting maladaptation, where current plant adaptations—whether natural or human-induced—may become detrimental under future climate scenarios (Lee et al. 2024). While the traditional view emphasized slow adaptation through the accumulation of small genetic changes, newer research suggests that larger, more abrupt changes in the genome’s structure might also be important drivers of the local adaptation process under environmental pressure (Todesco et al. 2020; Whiting et al. 2024). Abiotic stresses can induce genome instability in plants. The scale of genomic instability varies from alteration of the chromatin structure to modifications that result in changes in the expression of specific genes.

Identifying evolutionary responses is complex, requiring multiple generations and specific environments. While new alleles drive adaptation, focusing solely on the genome is limiting, as epigenetic modifications, symbiotic associations, and behavioral changes also contribute (Kelly 2019). The genomic mechanisms contributing to the adaptive variation include large-scale recombination events, chromosome deletions or chromosome fragment loss, transposable elements (TEs) mobilization, and heterochromatin alterations. Structural variants (SVs), including copy number variations (CNVs), can substantially alter gene regulation and expression, leading to phenotypic changes. CNVs have been linked to genes involved in responses to environmental stress in cultivated and wild species. CNVs in stress response pathways could have facilitated preadaptation to novel environments during crop dispersal (Zmienko et al. 2014).

The genomic resources providing the variability required to withstand stress are comparable, to some extent, in both animals and plants (Bennett et al. 2021; Fitzpatrick and Edelsparre 2018). Environmental and physiological stress affect mutational processes in *Drosophila* and *Caenorhabditis* species (Saxena et al. 2019). The contribution of structural variants to adaptive evolution remains largely unexplored (Rech et al. 2019). In *Drosophila suboscura*, chromosomal inversion frequencies are related to latitude and influenced by seasonal changes (Eric et al. 2023; Zivanovic et al. 2021): global warming induces the replacement of high-latitude by low-latitude chromosomal inversion frequencies (Santos et al. 2023). One specific inversion (O7), which exerts effects on reproduction and immunity, shows a seasonal cycle that rises with heat waves. Facing high extinction risk from extreme events, *Drosophila* species on volcanic islands show adaptive speciation, potentially dependent on stress-triggered TE activity (Craddock et al. 2016). In *D. melanogaster*, transposons move to nearby genes involved in stress response; for example, it was proven that heat, but not UV-C, can promote transposition of the TE *mariner-Mos1* (Jardim et al. 2015). In addition, they affect some behavioral genes that could act as “first responders” during climate change adaptation. In *Caenorhabditis*, the *Heat Shock Elements* (*HSE*s) interact with the *Heat Shock Factors* (*HSFs*) to mediate the transcriptional response of the *HSF* target genes (Zhao et al. 2020). In the same species, Helitrons transpose to *HSEs*, according to a species- and race-specific way (Pappalardo et al. 2021).

Animal genomic plasticity may be insufficient for adaptation to changing environments. In yellow warblers, a mismatch between genomic and environmental variation has been noted, with highly mismatched populations potentially facing decline (Bay et al. 2018). It is also known that some of the animal genes involved in the response to environmental changes are epigenetically regulated, and this affects food-searching and variation in exploratory strategies (Bay et al. 2018; Fitzpatrick and Edelsparre 2018).

## Disentangling phenotypic and genetic plasticity

Disentangling the roles of phenotypic plasticity and genetic adaptation in how plant populations respond to climate change is a significant challenge (Bernatchez et al. 2024). The primary difficulty lies in quantifying what proportion of an observed trait shift is a flexible, plastic response to environmental cues, which can obscure the underlying genetic changes driving adaptation. To overcome this, researchers use several methods to isolate genetic contributions from environmental effects."Common garden"experiments are a foundational approach where different populations are grown together under identical conditions. This setup removes environmental variables, ensuring that any observed differences between the populations are due to their genetic makeup. Reciprocal transplantation assesses adaptation by moving individuals between their native and different environments (Franks et al. 2018). This method is particularly effective at revealing the specific interactions between an organism’s genes and its environment. Resurrection ecology provides a temporal view by comparing ancestral populations with their modern descendants to track evolutionary changes that have occurred over time (Benning et al. 2023). These experimental approaches, often conducted in natural field settings and supplemented with demographic data and simulations, are crucial for accurately assessing rapid adaptation to climate change.

Local adaptation research focused on simple fitness traits, leaving the genotype-environment-fitness interplay understudied. Genes showing differential expression and divergent selection, likely crucial for adaptation, are identified via GWAS, QTL mapping, and population/landscape genomics. Genomic scans identify genes with differential expression and adaptive divergence, elucidating the link between phenotypic plasticity and evolutionary adaptation (Rellstab et al. 2015). In *Brassica rapa*, comparing pre/post-drought genomes showed adaptation, potentially via allele frequency shifts (Franks et al. 2016). In Arabidopsis, GWAS associate SNPs and the environment of landrace origin; the scope was to predict the existence of phenotypic variation for adaptive traits (Franks and Hoffmann 2012). In *Welwitschia mirabilis*, it was described that extreme drought stress could have caused the bursts of junk DNA, subsequently silenced by methylation (Wan et al. 2021).

Landscape genomics (LG) studies environmental drivers of genetic variation across landscapes, investigating genomic changes for local adaptation and predicting climate change responses. It uses FST outlier tests to identify genetic regions with allele frequency differences and Genotype-Environment Association (GEA) to detect genomic regions/genes linked to adaptation by analyzing allele frequency-environment correlations. In oak, LG showed that local conditions affected DNA diversity of climate change response genes, facilitating future local adaptation studies (Meger et al. 2024). Unexpectedly, genomic responses of putatively climate-linked loci in the high Andean wetland plant *Carex gayana* did not significantly correlate with environmental variables (Bertin et al. 2020). This result supports a genome-wide genetic approach using FST.

Identifying specific genes for allelic studies remains challenging, even though allele frequency shifts can indicate climate change adaptation. Traditional approaches often target genes with known phenotypic effects or selection signatures. For instance, in red spruce, the study of local adaptation led to identifying candidate genes for drought tolerance, cold hardiness, and plant phenology (Capblancq et al. 2023).

The absence of phenotyping in GEA approaches allows for a simplified approach to discovering stress-related alleles, even if the traits considered are cryptic in physiological terms. Most GEA methods use linear models to test genetic-environment relationships (nonlinear methods are also available)(Lasky et al. 2023). Machine learning has expanded the utility of GEAs by enabling predictions of adaptive alleles under different climate scenarios and by identifying vulnerable cultivar-locality combinations. While GEA studies have primarily focused on wild species, their application to crop species is promising, particularly for landraces adapted to local conditions (Gao et al. 2023; Lasky et al. 2023). In sorghum landraces, GEA identified numerous SNPs associated with environmental features, that could forecast differential responses to experimentally generated drought stress and aluminum toxicity (Lasky et al. 2015). In the wild sunflower, GEA detected structural variants underlying resistance to drought and heat (Todesco et al. 2020).

GEA data assesses genomic vulnerability by estimating poorly adapted species’ performance under climate change. Genomic vulnerability reflects the extent of genomic change needed to keep pace with climate change, typically assessed by comparing allele frequencies across populations in diverse climates. If local adaptation exists, populations are expected to possess alleles of selected loci with optimized frequencies in their specific conditions. Linking variants (e.g., SNPs) to climate drivers requires comparing populations geographically, showing correlations with climate variables like temperature extremes or rainfall variability (Hoffmann et al. 2021).

By identifying specific genes or loci associated with adaptive traits, researchers can determine the genetic contribution to a phenotype, which is the fundamental step in distinguishing it from a purely plastic, environmental response (Anderson and Song 2020). This approach associated SNPs between drought-tolerant and sensitive *Melilotus ruthenica* accessions with hundreds of candidate genes for drought tolerance (Wang et al. 2021c). The genes involved show variation in coding and cis-regulatory regions. For example, in *Mimulus guttatus*, there is divergence in promoter regions of genes differentially expressed between habitats, possibly leading to modification of binding sites for transcription factors, and altering the environmental sensitivity of gene expression (Takou et al. 2024; Yu et al. 2024; Zhang et al. 2024).

Pangenome analyses are key to understanding plant genotypic plasticity by decoding otherwise unexplored genomic variation, including the presence or absence of loci (SVs)."Dispensable"genes, non-essential under standard conditions, play a crucial role in adaptation and evolution. They are frequently enriched for functions related to biotic and abiotic stress tolerance, across species like soybean, tomato, wheat, rice, and brassicas (Fuentes et al. 2019). These findings suggest that the gene repertoire varies greatly among ecotypes and that gene or allele variation in each ecotype provides a basis for local adaptation (Kang et al. 2023). An example is the *Sub1* locus of rice: flooding-tolerant varieties have the *Sub1A* gene (on top of *Sub1B* and *Sub1C*), while susceptible varieties lack this locus (Xu et al. 2006).

An overview of recent examples of methods used to investigate genomic plasticity under climate change is reported in Table 1.

**Table 1:** Recent examples of methods analyzing genomic plasticity for climate change adaptation, and the current state of breeding in crop plants

Level of intervention	*Arabidopsis thaliana*	Rice	Maize	Wheat	Tomato
Genomic plasticity under stress	Epigenomic profiling, chromatin accessibility assays, stress-induced TE activation, GWAS & GEA at plasticity loci	Pan-genome analysis, GWAS & GEA at plasticity loci	Stress-induced TE activation, single-cell transcriptomics, GWAS & GEA at plasticity loci, landscape genomics	Epigenomic profiling, landscape genomics	Alternative splicing analysis, GWAS & GEA at plasticity loci
Understanding the mechanisms of plastic response	Chromatin topology changes, ChIP-seq of stress-responsive TFs, CRISPR screens, plasticity QTLs mapping, environmental genome-wide association	RNA modifications, miRNA profiling, transcriptomic meta-analysis	TE expression dynamics, chromatin topology changes, RNA-directed DNA methylation	Non-coding RNA and plasticity, stress-induced splicing, QTL x Environment Interactions (QEI)	Chromatin topology changes, eQTL
Induction of plasticity variability	Transgenerational epigenetic variability, targeted epigenetic editing	Induced hybrid epigenomes	TE activation breeding, mutation breeding	Doubled haploids, interspecific crosses	Longitudinal GWAS under stress
Breeding of climate-resilient crops		MAS, epigenetic-assisted selection, speed breeding	Breeding based on GEA-informed markers, GS breeding, multi-omics prediction models	MAS, hybrid breeding, haplotype-based GWAS	MAS, introgression of heat-resilient epialleles

*GWAS* genome-wide association studies; *GEA* genome-environment association; *TE* Transposable Element; *QTL* Quantitative Trait Locus; eQTL: expression Quantitative Trait Locus; ChIP-seq: Chromatin Immunoprecipitation sequencing; TF: Transcription Factor; GS: Genomic Selection; MAS: Marker Assisted Selection. Literature supporting Table 1 is reported as Online material

## Polyploidization and climate change

Fourteen Whole Genome Duplications (WGDs) occurred in angiosperms during periods of global changes and mass extinctions (Koenen et al. 2021). Particularly relevant were cooling periods at the Cretaceous-Tertiary boundary, during the Triassic-Jurassic transition (the fourth major extinction event), and at the Permian –Triassic transition (the third extinction) (Carretero-Paulet and Van de Peer 2020; Koenen et al. 2021; Van de Peer et al. 2021; Wu et al. 2020). Polyploidization confers adaptability to environmental variability (Xu et al. 2018) because it is associated with changes in gene expression, genomic shock, and epigenetic remodeling (Van de Peer et al. 2021). WGD facilitates evolutionary rescue under stress by expanding phenotypes and adaptive variation, enabling better population responses to selection (Ebadi et al. 2023; Yu et al. 2024).

In stressful environments, the Genetic Regulatory Networks (GRNs) alterations or disruptions may offer variants essential for survival. Polyploidy favors species adaptation via differential modulation of duplicated genes (Su et al. 2021). Following major challenges after species extinction, genes controlling plant responses to cold temperatures and decreased sunlight were more likely retained after WGD.

Polyploid genome complexity impacts chromatin organization, but genome structure dynamics during polyploidization are poorly understood. Polyploids likely gained a selective advantage from novel traits, possibly originating from genes’ subfunctionalization and separate evolution. Their specific structural variants in gene regions might be critical for adapting to future climate change (Hamala et al. 2024). Drastically changed environments likely reduce competition and open new niches, possibly explaining the past rapid spread of invasive species with doubled genomes (Birchler and Yang 2022). It is important to distinguish the adaptive roles of autopolyploidy and allopolyploidy. Autopolyploidy enhances stress tolerance by amplifying the existing genetic potential of a single species through increased gene dosage and allelic diversity. In contrast, allopolyploidy provides a more immediate and versatile adaptive advantage by combining the distinct, pre-adapted genetic toolkits of two different parental species (Van de Peer et al. 2021).

Polyploidization generally provides a buffering effect by tolerating deleterious mutations, unfit gene expression, or epigenetic remodeling. This may explain the higher prevalence of polyploid species at poleward latitudes and higher elevations previously covered by ice sheets (Rice et al. 2019). A similar pattern is seen in contemporary invasive species like *Spartina anglica*, which uses allopolyploidy to adapt to fluctuating salt marsh conditions (Giraud et al. 2021). Research on terrestrial plants generally indicates a correlation between dry environments and polyploidy, suggesting polyploid species are more common in arid habitats than their diploid relatives (Van de Peer et al. 2021). Switchgrass shows genomic evidence of climate adaptation. As a polyploid, its subgenomes likely provided genetic variability used by natural selection to increase adaptability (Lovell et al. 2021). Resequencing grasses subfamilies identified a specific pattern of differential retention/loss, termed PROSOL (Pair Retained in One lineage but Single-copy in Other Lineages). Some genes/alleles involved in abiotic stress response bind to stress-related gene promoters, modulating dehydration tolerance. The Poaceae family underwent WGD, and the resulting duplicate genomes show differential gene retention among subfamilies, including those supporting environmental adaptations (Zhang et al. 2024). In Oryzoideae, the *ACOT* gene might aid osmotic stress response by increasing wax deposition and regulating root transport. This subfamily has two *ACOT* paralogues, while others conserve only one copy (Zhang et al. 2024). In wheat, *CK2* is involved in light-signal transduction and salicylic acid-mediated defense. RNA-seq data revealed distinct expression patterns of *CK2β* paralogues under cold stress, suggesting their role in Pooideae species’ adaptation to diverse environmental conditions (Zhang et al. 2024).

TEs are linked to polyploidization. In tetraploid rice, increased stress-responsive gene expression can induce hypermethylation and suppression of nearby TEs. The hypothesis suggests a relationship between hypomethylation due to polyploidy and stress response, where hypermethylation limits TEs activity near stress-response genes (Wang et al. 2021b).

From a morphological and physiological standpoint, the connection between polyploidy and a plant’s tolerance to stress is multifaceted. Potential advantages for polyploids include physical traits like thicker leaves that improve water retention during droughts. Polyploid advantages might include thicker, water-retaining leaves during droughts (e.g., *Betula platyphylla*), fewer stomata, and different pit anatomy (Guo et al. 2016). In cases, the diploids show less flexibility in responding to drought stress (Yoo et al. 2024). Plant hormones are possibly involved: tetraploids have more ABA, conferring resistance to drought in *Lycium ruthenicum* (Rao et al. 2020). Moreover, WGD may lead to changes in water use efficiency, photosynthetic rate, phenology, transpiration (Sessa 2019), as exemplified in the tetraploid *Arabidopsis thaliana*, in the tetraploids *Oryza sativa* and *Citrullus lanatus*, which are more tolerant to salt and drought stress than the diploid (Yang et al. 2014; Zhu et al. 2018). Overall, polyploidy’s effect on stress tolerance is generally considered positive, but depends on the specific plant and stress type.

## Endopolyploidy

Endopolyploidy is a state where cells replicate their DNA without division, which serves as a strategy for some plants to adapt to environmental changes. For example, Arabidopsis plants in colder environments show higher degrees of endopolyploidy (Pacey et al. 2020; Zedek et al. 2020), a condition that enhances salt tolerance but can lower fitness under non-saline conditions. In terms of drought survival, endopolyploid leaves may have an advantage as the increased intracellular storage could mean they contain more chloroplasts or a higher water content. However, endopolyploidy can also have a downside; under saline conditions, the larger cell volume may reduce the photosynthetic rate and decrease salt tolerance (Corneillie et al. 2019). Endoreduplication is the process where specific cells within somatic tissues replicate their DNA without dividing. This is a common developmental strategy in plants, occurring in tissues like leaves, fruit flesh, and root hairs to increase cell size and metabolic output. While the endopolyploid state of a parent’s leaf is not inherited, the genetic tendency to produce endopolyploid leaves is. Endoreplication in plants may occur in response to abiotic stresses: increasing gene copies increases passive and active defense against stresses (Kolodziejczyk et al. 2023).

## Gene copy number variation (CNV)

Polyploidization disrupts mitotic fidelity, increasing rates of unequal crossing over, replication errors, and chromosome missegregation—key drivers of CNV under climate stress response (Lye and Purugganan 2019; Mareri et al. 2020). *HSFs* and *HSP* genes are critical for climate adaptation and vary in copy number across plant species. Lettuce has three times more *HSP70* family genes than Arabidopsis and twice as many as rice, due to genomic tandem duplications (Kim 2021). In *A. thaliana,* the perturbation of *HSP90* increases somatic homologous recombination, possibly leading to genome instability (Weinstein et al. 2019). Duplicated gene copies can be differentially regulated. For example, in rice, only one *SD1* gene copy promotes internode elongation in deep water via gibberellin biosynthesis, suggesting neofunctionalization. This adaptation may have contributed to rice thriving in periodically flooded conditions. Another example is the *AP2/ERF* gene subfamily, crucial for stress responses. Its expansion is influenced by whole-genome duplication, tandem duplication, and transposition events in various plant species. These duplications may have led to further copy number variation and functional divergence within the subfamily (Zhu et al. 2024). CBF gene variations in copy number, linked to winter hardiness, were selected during temperate cereal domestication. Coordinated *VRN-1/CBF* gene regulation is essential for frost resistance in cereals (Mareri et al. 2020). The *HKT1* gene has experienced repeated tandem duplications during adaptation to saline environments in the desert poplar (Ali et al. 2019). Not always is the adaptive nature of the genomic changes linked to mutation in candidate genes: in *Fagus sylvatica*, several anonymous SNPs have been associated with climatic gradients (Capblancq et al. 2020). The Neolithic spread of agriculture into northern Europe led to adaptations in wheat (*Triticum aestivum*), particularly to daylength and vernalization, controlled by the *VrnA1* gene. As wheat expanded north, an increase in *VrnA1* gene copy number, leading to a higher vernalization requirement, was observed. Geographically structured patterns of *VrnA1* copy number demonstrate how gene duplication facilitated wheat’s adaptation to novel environments (Wurschum et al. 2015).

## Plant transposons and stresses

Once seen as genomic parasites, TEs are key drivers of genome evolution, especially when activated by environmental challenges like abiotic stress or UV (Liang et al. 2021a; Negi et al. 2016). TEs influence genome structure as a source of genetic variation. They can induce phenotypic variation through insertional mutagenesis, modify gene function by inserting into cis-regulatory regions, or create novel genes, all leading to adaptive traits and speciation (Chuong et al. 2017; Joly-Lopez and Bureau 2018). Environmental factors influence retrotransposons’ transcriptional activity and subsequent genomic mobilization (Dubin et al. 2018).

In plants, genome-wide methylation patterns often coincide with high TEs density, suggesting methylation’s role in silencing TEs and preventing damage from their uncontrolled activity (Hassan et al. 2023). Retrotransposon transcription and Long Terminal Repeat (LTR) activity directly influence neighboring gene expression by producing antisense and sense RNAs. This mechanism contributes to allelic variation in gene expression patterns under stress. In maize, approximately 20% of stress-responsive genes harbor TEs within their promoter regions. In sunflower, LTR-retrotransposons are activated by phytohormone-induced abiotic stress, and in poplar, the same are triggered by drought, cold, and heat (reviewed in (Papolu et al. 2022). In soybean, the adaptation to high altitudes is correlated with TEs disruption of the *Phytochrome A* gene (*GmphyA2*) (Tsubokura et al. 2013). *ONSEN,* an Arabidopsis *Ty1/Copia* retrotransposon, inserts into new genomic locations when activated by heat stress (Ito et al. 2016). Mangrove genomes are smaller than terrestrial angiosperms due to TE suppression and gene loss. Environmental stress accelerates mangrove genome instability, but adaptive TE loss reduces DNA replication costs and prevents further instability (Lyu et al. 2018).

In TE exaptation (ETE), TEs gain new, unrelated functions but retain sensitivity to inducing conditions. This functional versatility aids their persistence in the genome without original selective pressures (Joly-Lopez and Bureau 2018; Joly-Lopez et al. 2017). The mechanisms that lead to ETE may include epigenetic desilencing under stress and genomic reorganization, such as polyploidization (Vicient and Casacuberta 2017).

Families of transposons, like the Helitrons, are known to recruit and dispatch fragments of host genes, thus accelerating phenotypic variability and increasing the chances for TEs to express their genes (Pimpinelli et al. 2019; Wang et al. 2022). In poplar, Helitrons control the expression of stress-response genes (Zhao et al. 2022). In heat-stressed wheat, chromatin status affects *HSE-HSF* interaction. Helitrons influencing HSE positioning could alter this interaction, impacting stress-response gene regulation (Liu et al. 2018b).

TEs can evolve into lncRNA genes, which participate in the stress response (Pimpinelli et al. 2019; Urquiaga et al. 2020). TEs influence gene expression and stress tolerance by producing siRNAs. Dehydration can enhance retrotransposon transcription, increasing genomic insertion frequency. This can integrate retrotransposons into regulatory elements, promoting their transcription and siRNA amplification. This positive feedback loop may contribute to desiccation tolerance even without direct selection. In the resurrection plant *C. plantagineum*, stress-induced *CDT-1* siRNA promotes desiccation tolerance by regulating ABA/dehydration genes. This illustrates genome-environment interaction where dehydration boosts siRNA levels and tolerance via transcription/transposition (Negi et al. 2016). In maize, a *Gypsy* retrotransposon is located within a gene associated with drought tolerance (*ZmPPC2C16*). Under drought conditions, the production of siRNAs from the Gypsy TE is enhanced and these are likely to regulate the expression of genes involved in drought tolerance, including *ZmPPC2C16* itself (Sun et al. 2023). In *Arabidopsis*, stress-induced activation of *Athila* retrotransposons leads to siRNAs that can negatively regulate stress response genes. For example, *Athila*-derived siRNAs repress the stress-induced expression of *UBP1b*, highlighting the potential for transposable elements to disrupt adaptive processes (Slotkin 2010).

While TEs can contribute to adaptive evolution, they can also be harmful. Heterochromatinization, a process that condenses chromatin around TEs, mitigates these threats by silencing them.

## Stresses and somatic mutations

Plants’ late determination of embryonic germline cells significantly impacts somatic mutation fate. Key details include low somatic mutation frequency, a positive correlation between mutation rate and intergenerational transmission, and the influence of plant architecture on mutation distribution, as plant structure dictates mutation origin, accumulation, and inheritance. Plant somatic mutations can potentially affect reproductive organs despite occurring in non-reproductive cells (Reusch et al. 2021). The traditional view posits random mutations, including somatic ones, without adaptive intent. However, recent evidence indicates substantial heterogeneity in mutation rates across the genome, influenced by factors like transcription-coupled repair and local epigenetic modifications. Furthermore, mutations are not random and preferentially occur in expressed sequences (Monroe et al. 2022). Elevated mutation rates under stress should accelerate adaptive evolution. However, whether plastic mutation responses in plants provide adaptive benefits has not been systematically tested (Choi et al. 2021). Salinity stress causes mutations/epimutations in Arabidopsis; other stresses (e.g., UV-C) enhance homologous recombination, increasing genetic variability and potentially enabling adaptive phenotypes (Georgieva and Vassileva 2023). Such phenomena are more evident in perennial species (Ban and Jung 2023): in *Prunus mira* trees living on the Tibet plateau, the genes involved in DNA repair and response are overrepresented (Li et al. 2021). Environmental stresses can also affect the intragenomic variability of the mutation rate (Lu et al. 2021; Zhu et al. 2021). For example, microsatellite instability in somatic cells is affected by exposure to UVC, heat, and cold stresses (Yao and Kovalchuk 2011).

Stress raises mutation rates (by impairing repair or accumulation). Although stress may change positive variant patterns, increased mutation/epimutation frequencies also raise the burden of deleterious variants (Belfield et al. 2021; Cruzan et al. 2022; Monroe 2023). Whether stress-induced mutation rate correlates with mutation frequency in stress-counteracting genes is unknown, and the evolutionary role of somatic mutation remains largely unexplored (Monroe et al. 2022). Multigenerational Arabidopsis mutation accumulation lines were exposed to extreme and moderate heat stress. Heat significantly increased mutations, especially indels and SNPs, increasing transitions and transversions mainly in intergenic, genic, and TE-rich regions, and promoted mutation accumulation in genes related to defense, DNA repair, and signaling pathways. Additionally, methylation at mutation sites increased. Notably, mutation types and distribution in individual plants/populations differed between extreme and moderate heat, suggesting stronger selection in populations (Dubrovina and Kiselev 2016; Lu et al. 2021).

Chromatin states associated with active/silent genes may cause differential mutation rates. Histone modifications influence DNA repair, shaping mutation patterns. Stresses altering chromatin structure via methylation or histone modifications result in altered homologous recombination frequency (Yao and Kovalchuk 2011). A hypothesis suggests that cold tolerance genes have epigenomic states with lower mutation rates in cold environments. In warm environments, the silenced gene may have higher mutation rates due to increased transposon insertion. Disrupting this gene could even improve fitness, as its aberrant expression might be harmful without cold stress. An example is *CBF2* (Monroe 2023).

Structural variation (SV) is important in adaptation by modulating gene expression. For example, in *A. thaliana,* a paracentric inversion has been associated with fertility under drought conditions (Fransz et al. 2016). In cucumber, deleted genes were associated with histone methylation and abiotic stress response, while duplicated genes were often involved in the reproductive process (Zhang et al. 2015). Recent evidence suggests telomeres control flowering time and, indirectly, plant capacity to cope with adverse environments. Natural telomere length varies, with temperate plants having the longest telomeres (Choi et al. 2021).

## Role of chromatin remodeling and plasticity in plant stress responses

Climate change is believed to induce heritable epigenetic changes, offering an alternative evolutionary route (Mozgova et al. 2019). In eukaryotes, chromatin structure maintains genome stability and structural flexibility, both crucial for organisms facing environmental stresses (Probst and Mittelsten Scheid 2015)(Santos et al. 2020). Constantly modified and rearranged during DNA replication, transcription, and repair, dynamic chromatin regulation enables rapid adaptation (Kim 2021). Histone modifications and remodelers influence local chromatin architecture, regulating nucleosome positioning and accessibility, thereby affecting gene expression globally and at specific loci. Environmental effects on gene expression often involve changes in cytosine methylation, histone variants, and histone modifications. These chromatin modification changes affect various responsive loci, including 5mC and 6mA DNA methylation genes. Stressed plants prioritize survival by shifting metabolic energy from protein translation and cell elongation to chromatin modifications, including increased histone acetylation at promoters and stress-responsive genes, and DNA demethylation for target gene derepression (Nunez-Vazquez et al. 2022). We focus on chromatin remodelers, which dynamically modify chromatin architecture, enabling regulatory protein access to DNA through covalent histone modifications and ATP-dependent nucleosome reorganization. Chromatin remodeling generates nucleosome-free regions, facilitating DNA repair at damage sites and transcription at gene promoters (Bornelov et al. 2018). Under various stresses, chromatin remodelers activate chromatin, crucial for transcriptional regulation and DNA repair during stress (Wang et al. 2024).

Heat stress alters chromatin structure, promoting transient promoter-enhancer interactions that activate heat-responsive genes, contributing to thermotolerance (Kumar and Rani 2023; Liu et al. 2015). The causal link between chromatin remodeling and stress-induced transcriptional changes in plants has been explored. Arabidopsis methylome analysis revealed a 45% increase in CG methylation, more frequent in genic regions, in stressed lineages versus controls. Most of these changes were stable across generations, but some were lost. In *Populus trichocarpa*, under water stress, the proportion of methylated cytosines was 10% compared to 7.7% in the well-watered plants (Akhter et al. 2021). In Arabidopsis, *H3K9/H3K14* acetylation enhances thermotolerance and salt tolerance by activating heat/salt stress genes like *HSFA3*, *UVH6*, *CTL1*, *PGX3*, and *MYB54*. In *Brassica rapa*, heat, drought, and salinity stress correlate with methylated regions and differentially expressed genes (Liu et al. 2018a). In rice, changes in chromatin structure are linked to DNA accessibility and variations in gene expression (Liang et al. 2021b). Again, comparative methylome analysis in rice revealed differential methylation patterns associated with stress-responsive genes (Garg et al. 2015). Hypermethylated regions in this species showed reduced gene expression and increased small RNA abundance, suggesting a complex interplay between DNA methylation and small RNA silencing (Banerjee and Roychoudhury 2017; Ding et al. 2022). In *Medicago sativa*, a potential role for DNA demethylation in drought tolerance mechanisms is hypothesized (Ventouris et al. 2020). The alpine *Ranunculus* shows, in response to cold, a mark of the epigenetic impact of the genome doubling, which results in phenotypic plasticity (Huang et al. 2023). UV-B controls Arabidopsis photomorphogenic genes and causes photodamage. Irradiation boosts heterochromatin content and promotes varying heterochromatin dynamics among acclimated ecotypes (Johann To Berens et al. 2023). DNA methylation helps control TE activation. Mitotically stable heterochromatinization ensures TEs remain silenced across plant cell divisions (Hassan et al. 2023).

Drought stress induces lncRNAs in maize tissues, suggesting a role in adaptation. Natural Antisense Transcripts (NATs)—regulatory RNAs among lncRNAs—are notable in maize lines with extreme positive and negative drought responses. Despite unclear functions in plant stress, stress-associated NATs show significant hypomethylation and fewer transposable element sequences than non-NAT genes, suggesting unique regulatory mechanisms for drought tolerance (Lv et al. 2019).

Plants exhibit stress memory, i.e., enhanced tolerance to subsequent stress. Histone methylation and heterochromatin decondensation are implicated in transcriptional memory of stress genes (Baurle and Trindade 2020). *FLC* repression during cold response involves spatial reorganization of chromatin loci. Cold stress response genes are physically grouped within the nucleus, enhancing gene expression efficiency and coordination. Cold-induced *FLC* repression involves both epigenetic and significant spatial chromatin reorganization within the nucleus (Rahman et al. 2024). *JMJs* demethylases maintain stress memory and enable *Arabidopsis thaliana* heat acclimation by inducing demethylation of *HSP22/HSP17.6C*, allowing *HSP* gene activation that persists after heat stress, proving an epigenetic memory mechanism (Yamaguchi et al. 2021). A similar situation occurs upon acclimatization after heat stress in Arabidopsis, where chromatin remodeling is governed by the interaction of *BRM*/*CHR11*/*17* chromatin remodelers and *FORGETTER 1* (*FGT1*), causing the sustainable induction of the heat-responsive genes *HSA32* and *HSP18*.*2/22.0* (Willige et al. 2021).

## Alternative splicing

Alternative splicing (AS), an RNA-level plasticity enhancing plant genetic flexibility and adaptability, involves conserved core events like exon skipping, intron retention, and alternative splice sites in plants. 61% of Arabidopsis thaliana genes undergo alternative splicing. Plant research focuses on intron retention coupled with Nonsense-Mediated Decay (NMD) to regulate gene expression by degrading defective mRNAs, potentially aiding stress tolerance by reducing protein synthesis metabolic cost via NMD triggered by premature termination codons in retained introns. Key stress response genes frequently undergo alternative splicing, generating multiple transcript isoforms, while counterintuitively, non-intron retention alternative splicing, like exon skipping, enriches stress-responsive genes and significantly regulates expression through NMD and upstream open reading frames (Kim et al. 2024).

When compared to animals, plants exhibit a disproportionate reliance on AS for stress responses (Martin et al. 2021). In Arabidopsis, a truncated *HSFA2* splice variant autoregulates by binding its promoter to stimulate transcription, unlike the full-length form. *DREB2* regulates *HSFA2* and other *HSFs* and undergoes stress-responsive alternative splicing across plant species, where normally *DREB2B* produces a non-functional transcript, but stress significantly increases full-length functional transcripts (Capovilla et al. 2018; Roces et al. 2022). Flowering time, a plastic and genetically controlled trait under a changing climate, is partly fine-tuned by genomic structural variation and alternative splicing. In Arabidopsis, a spliced FLM isoform regulates flowering based on temperature by interacting with splicing factor *SF1*, enabling temperature-responsive flowering fine-tuning (Lee and Adams 2020).

Other genes for resilience to adverse climate undergo alternative splicing under stress, such as *bZIP60* mRNA spliced by heat-induced ER stress. Arabidopsis *ZIFL1* alternative splicing generates two distinct protein isoforms with different subcellular locations and functions: full-length *ZIFL1* is a tonoplast auxin transporter, while the truncated form moves to the nucleus to activate protein homeostasis genes and is also a plasma membrane protein crucial for stomatal regulation and drought tolerance. Most Arabidopsis serine/arginine rich protein genes, critical splicing regulators also undergo alternative splicing, exhibit altered splicing patterns in response to various environmental stresses (Martin et al. 2021).

Splicing memory, the cell’s capacity to retain splicing history influencing future events, is conserved across taxa and aids adaptation to changing environments (Liu et al. 2022). A model suggests epigenetic/splicing mechanisms maintain acclimation-associated splicing patterns long-term, possibly leading to transgenerational effects (Roces et al. 2022).

## Conclusions

Genomic vulnerability to climate change occurs when plant populations lack the genetic diversity needed to adapt, which can cause them to decline. This risk is especially high if they do not have access to alleles adapted to warmer conditions, such as those found at lower latitudes and elevations. Human-induced climate change may outpace plant genomic adaptation. A key question is whether populations adapt via existing genetic variation, gene flow, or new mutations. Advancements in evolutionary and landscape genomics allow distinguishing climate and spatial factors shaping genetic variation. The identification of biologically significant shifts in allele frequencies is challenging, although researchers often focus on loci influencing phenotypes/under selection and linked neutral loci. Genome scanning targeting candidate or anonymous loci is another approach, though their link to adaptive changes is often unclear. Understanding selection and spatial isolation’s influence on genomic variation is crucial for assessing adaptation’s role in diversification and identifying climatic selective pressures, a complex task due to overlapping factors. In conclusion, plant genomic plasticity is key for adapting to climate change, highlighting the interplay between environment and genetic potential. Plants adapt to diverse abiotic stresses like heat, drought, and salinity using a shared toolkit of genomic and epigenetic mechanisms. Ploidy changes confer broad tolerance through advantageous"gigas effect"morphologies that improve water conservation under heat stress (Porturas et al. 2019) and enhance water-use efficiency during drought. In saline environments, polyploidy also provides genetic buffering that improves ion homeostasis and osmotic adjustment (Song et al. 2025). Transposons, activated by stress, drive adaptation by creating novel genetic variation. They rewire gene networks in response to heat (Nguyen et al. 2025), generate selectable traits like deeper roots for drought tolerance (Wang et al. 2021a), and alter the expression of key genes like ion pumps to combat salinity (Ito et al. 2016). Epigenetic responses offer dynamic and fine-tuned control. Chromatin remodeling, via H2A.Z histone eviction and histone acetylation, rapidly activates defense genes under heat and salinity stress (Kan et al. 2023; Yung et al. 2021). Alternative splicing further refines the adaptation by generating functionally diverse protein isoforms. This process creates a regulatory"memory"for acquired thermotolerance (Kan et al. 2023), modulates the ABA signaling pathway to control stomata and root development under drought (Liu et al. 2022), and precisely regulates ion homeostasis to cope with salinity (Alhabsi et al. 2025), ensuring a balanced and stress-specific response.

Understanding these mechanisms enhances knowledge of plant resilience and informs the development of climate-resilient crops via breeding and biotechnology. Based on the findings, the mechanisms of genomic plasticity can be harnessed to enhance crop resilience through applied plant breeding. Modern genomic tools, such as GWAS, GEA, and pangenome analyses, allow for the precise identification of genes, structural variants, and alleles that confer tolerance to abiotic stresses like drought and salinity. This information can be directly integrated into breeding programs via MAS and Genomic Selection (GS). Furthermore, understanding these processes opens the door for innovative strategies (Raza et al. 2024). For example, GEA-informed markers can guide breeding in maize, while epigenetic-assisted selection and the introgression of heat-resilient epialleles are being explored in rice and tomato, respectively. Further details are provided in Table 1. By leveraging the plant’s natural adaptive potential—from targeted epigenetic editing to TE activation breeding—these findings provide a roadmap for developing the next generation of climate-resilient crops, which is crucial for ensuring future food security. As climate change accelerates (Intergovernmental Panel on Climate 2023), further research into plant genomic responses is crucial for biodiversity, food security, and sustainable ecosystems.

## Competing interests

The authors have no relevant financial or non-financial interests to disclose.

## Supplementary Information

Below is the link to the electronic supplementary material.

Supplementary file 1 (DOCX 26 KB)
