# Supplementary material for: Climate change and plant genomic plasticity
Source: Theor Appl Genet. 2025 Aug 27;138(9):231. doi: 10.1007/s00122-025-05010-x (PMC12390881; doi:10.1007/s00122-025-05010-x)
Supplement: Supplementary file 1 — (DOCX 26 KB) [file 122_2025_5010_MOESM1_ESM.docx]

**The electronic supplementary material includes only the literature used to create Table 1 (which is reported here for the reviewer’s convenience).**

| **Level of intervention** | ***Arabidopsis thaliana*** | **Rice (*Oryza sativa*)** | **Maize (*Zea mays*)** | **Wheat (*Triticum aestivum*)** | **Tomato (*Solanum lycopersicum*)** |
| --- | --- | --- | --- | --- | --- |
| **Genomic plasticity under stress** | Epigenomic profiling, chromatin accessibility assays, stress-induced TE activation, GWAS & GEA at plasticity loci | Pan-genome analysis, GWAS & GEA at plasticity loci | Stress-induced TE activation, single-cell transcriptomics, GWAS & GEA at plasticity loci, landscape genomics | Epigenomic profiling, landscape genomics | Alternative splicing analysis, GWAS & GEA at plasticity loci |
| **Understanding the mechanisms of plastic response** | Chromatin topology changes, ChIP-seq of stress-responsive TFs, CRISPR screens, plasticity QTLs mapping, environmental genome-wide association | RNA modifications, miRNA profiling, transcriptomic meta-analysis | TE expression dynamics, chromatin topology changes, RNA-directed DNA methylation | Non-coding RNA and plasticity, stress-induced splicing, QTL x Environment Interactions (QEI) | Chromatin topology changes, eQTL |
| **Induction of plasticity variability** | Transgenerational epigenetic variability, targeted epigenetic editing | Induced hybrid epigenomes | TE activation breeding, mutation breeding | Doubled haploids, interspecific crosses | Longitudinal GWAS under stress |
| **Breeding of climate-resilient crops** |  | MAS, epigenetic-assisted selection, speed breeding | Breeding based on GEA-informed markers, GS breeding, multi-omics prediction models | MAS, hybrid breeding, haplotype-based GWAS | MAS, introgression of heat-resilient epialleles |

Acronyms used:

- *GWAS: Genome-Wide Association Studies*
- *GEA: Genome-Environment Association*
- *TE: Transposable Element*
- *QTL: Quantitative Trait Locus*
- *eQTL: expression Quantitative Trait Locus*
- *ChIP-seq: Chromatin Immunoprecipitation sequencing*
- *TF: Transcription Factor*
- *GS: Genomic Selection*
- *MAS: Marker Assisted Selection*

*Literature used to create Table 1.*

*Arabidopsis thaliana*

(Singh et al. 2024) (Chen et al. 2024) (Lin et al. 2024) (Kappel et al. 2023) (Alseekh et al. 2025) (Kappel et al. 2023) (Angon et al. 2023) (Napier et al. 2023) (Bogan and Yi 2024) (Harris et al. 2023) (Castellana et al. 2024) (Baduel et al. 2024)

*Oryza sativa*

(Azad et al. 2024) (Wei et al. 2024) (Duarte-Ake et al. 2023) (Junaid et al. 2024) (Mohanavel et al. 2024) (Bedford et al. 2023) (Cai et al. 2024)

*Zea mays*

(Sahito et al. 2024) (Wang et al. 2025) (Yang et al. 2023) (Bubb et al. 2024) (Fernie 2023) (Guarino et al. 2022) (Gao et al. 2023) (Cooper and Messina 2023) (Barreto et al. 2024) (Gunundu et al. 2023) (Djalovic et al. 2024) (Zhang et al. 2023)

*Triticum aestivum*

(Broccanello et al. 2023) (Babaei et al. 2024) (Alyahya and Taybi 2024) (Sun et al. 2022) (Li et al. 2024) (King et al. 2024) (Roychowdhury et al. 2024) (Gudi et al. 2024)

*Solanum lycopersicum*

(Guo et al. 2024) (Delarue et al. 2025) (Zhu et al. 2022) (Agho et al. 2024) (Hereil et al. 2024)

Agho C, Avni A, Bacu A, Bakery A, Balazadeh S, Baloch FS, Bazakos C, Čereković N, Chaturvedi P, Chauhan H, De Smet I, Dresselhaus T, Ferreira L, Fíla J, Fortes AM, Fotopoulos V, Francesca S, García-Perez P, Gong W, Graci S, Granell A, Gulyás A, Hidvégi N, Honys D, Jankovska-Bortkevič E, Jonak C, Jurkonienė S, Kaiserli E, Kanwar M, Kavas M, Koceska N, Koceski S, Kollist H, Lakhneko O, Lieberman-Lazarovich M, Lukić N, Luyckx A, Mellidou I, Mendes M, Miras-Moreno B, Mirmazloum I, Mladenov V, Mozafarian M, Mueller-Roeber B, Mühlemann J, Munaiz ED, Niedbała G, Nieto C, Niinemets Ü, Papa S, Pedreño M, Piekutowska M, Provelengiou S, Quinet M, Radanović A, Resentini F, Rieu I, Rigano MM, Robert HS, Rojas LI, Šamec D, Santos AP, Schrumpfova PP, Shalha B, Simm S, Spanic V, Stahl Y, Šućur R, Vlachonasios ΚE, Vraggalas S, Vriezen WH, Wojciechowski T, Fragkostefanakis S (2024) Integrative approaches to enhance reproductive resilience of crops for climate-proof agriculture. Plant Stress:100704, https://doi.org/10.1016/j.stress.2024.100704

Alseekh S, Klemmer A, Yan J, Guo T, Fernie AR (2025) Embracing plant plasticity or robustness as a means of ensuring food security. Nat Commun 16:461, https://doi.org/10.1038/s41467-025-55872-4

Alyahya N, Taybi T (2024) Transcriptome-wide characterization of alternative splicing regulation in Najran wheat (Triticum aestivum) under salt stress. Current Plant Biology 38:100334, https://doi.org/10.1016/j.cpb.2024.100334

Angon PB, Mondal S, Akter S, Sakil MA, Jalil MA (2023) Roles of CRISPR to mitigate drought and salinity stresses on plants. Plant Stress 8:100169, https://doi.org/10.1016/j.stress.2023.100169

Azad M, Tohidfar M, Ghanbari Moheb Seraj R, Mehralian M, Esmaeilzadeh-Salestani K (2024) Identification of responsive genes to multiple abiotic stresses in rice (Oryza sativa): a meta-analysis of transcriptomics data. Sci Rep 14:5463, https://doi.org/10.1038/s41598-024-54623-7

Babaei S, Bhalla PL, Singh MB (2024) Identifying long non-coding RNAs involved in heat stress response during wheat pollen development. Front Plant Sci 15:1344928, https://doi.org/10.3389/fpls.2024.1344928

Baduel P, De Oliveira L, Caillieux E, Bohl-Viallefond G, El Messaoudi M, Xu C, Barois M, Singh V, Sarazin A, Boccara M, Gilbault E, de France A, Quadrana L, Loudet O, Colot V (2024) 10.1101/2024.09.20.614076

Barreto CAV, das Gracas Dias KO, de Sousa IC, Azevedo CF, Nascimento ACC, Guimaraes LJM, Guimaraes CT, Pastina MM, Nascimento M (2024) Genomic prediction in multi-environment trials in maize using statistical and machine learning methods. Sci Rep 14:1062, https://doi.org/10.1038/s41598-024-51792-3

Bedford JA, Carine M, Chapman MA (2023) Detection of locally adapted genomic regions in wild rice (Oryza rufipogon) using environmental association analysis. G3 (Bethesda) 1310.1093/g3journal/jkad194

Bogan SN, Yi SV (2024) Potential Role of DNA Methylation as a Driver of Plastic Responses to the Environment Across Cells, Organisms, and Populations. Genome Biol Evol 1610.1093/gbe/evae022

Broccanello C, Bellin D, DalCorso G, Furini A, Taranto F (2023) Genetic approaches to exploit landraces for improvement of Triticum turgidum ssp. durum in the age of climate change. Front Plant Sci 14:1101271, https://doi.org/10.3389/fpls.2023.1101271

Bubb KL, Hamm MO, Min JK, Ramirez-Corona B, Mueth NA, Ranchalis J, Vollger MR, Trapnell C, Cuperus JT, Queitsch C, Stergachis AB (2024) The regulatory potential of transposable elements in maize. bioRxiv10.1101/2024.07.10.602892

Cai J, Shen L, Kang H, Xu T (2024) RNA modifications in plant adaptation to abiotic stresses. Plant Commun:101229, https://doi.org/10.1016/j.xplc.2024.101229

Castellana S, Triozzi PM, Dell'Acqua M, Loreti E, Perata P (2024) Environmental genome-wide association studies across precipitation regimes reveal that the E3 ubiquitin ligase MBR1 regulates plant adaptation to rainy environments. Plant Commun 5:101074, https://doi.org/10.1016/j.xplc.2024.101074

Chen B, Wang M, Guo Y, Zhang Z, Zhou W, Cao L, Zhang T, Ali S, Xie L, Li Y, Zinta G, Sun S, Zhang Q (2024) Climate-related naturally occurring epimutation and their roles in plant adaptation in A. thaliana. Mol Ecol 33:e17356, https://doi.org/10.1111/mec.17356

Cooper M, Messina CD (2023) Breeding crops for drought-affected environments and improved climate resilience. Plant Cell 35:162-186, https://doi.org/10.1093/plcell/koac321

Delarue M, Benhamed M, Fragkostefanakis S (2025) The role of epigenetics in tomato stress adaptation. New Crops 2:100044, https://doi.org/10.1016/j.ncrops.2024.100044

Djalovic I, Kundu S, Bahuguna RN, Pareek A, Raza A, Singla-Pareek SL, Prasad PVV, Varshney RK (2024) Maize and heat stress: Physiological, genetic, and molecular insights. Plant Genome 17:e20378, https://doi.org/10.1002/tpg2.20378

Duarte-Ake F, Us-Camas R, De-la-Pena C (2023) Epigenetic Regulation in Heterosis and Environmental Stress: The Challenge of Producing Hybrid Epigenomes to Face Climate Change. Epigenomes 710.3390/epigenomes7030014

Fernie AR (2023) On the role of transposons in balancing drought tolerance and yield. Trends Plant Sci 28:262-263, https://doi.org/10.1016/j.tplants.2022.12.005

Gao L, Kantar MB, Moxley D, Ortiz-Barrientos D, Rieseberg LH (2023) Crop adaptation to climate change: An evolutionary perspective. Mol Plant 16:1518-1546, https://doi.org/10.1016/j.molp.2023.07.011

Guarino F, Cicatelli A, Castiglione S, Agius DR, Orhun GE, Fragkostefanakis S, Leclercq J, Dobranszki J, Kaiserli E, Lieberman-Lazarovich M, Somera M, Sarmiento C, Vettori C, Paffetti D, Poma AMG, Moschou PN, Gasparovic M, Yousefi S, Vergata C, Berger MMJ, Gallusci P, Miladinovic D, Martinelli F (2022) An Epigenetic Alphabet of Crop Adaptation to Climate Change. Front Genet 13:818727, https://doi.org/10.3389/fgene.2022.818727

Gudi S, Halladakeri P, Singh G, Kumar P, Singh S, Alwutayd KM, Abd El-Moneim D, Sharma A (2024) Deciphering the genetic landscape of seedling drought stress tolerance in wheat (Triticum aestivum L.) through genome-wide association studies. Front Plant Sci 15:1351075, https://doi.org/10.3389/fpls.2024.1351075

Gunundu R, Shimelis H, Mashilo J (2023) Genomic selection and enablers for agronomic traits in maize (Zea mays): A review. Plant Breeding 142:573-593, https://doi.org/10.1111/pbr.13127

Guo Y, Shang X, Ma L, Cao Y (2024) RNA-Binding Protein-Mediated Alternative Splicing Regulates Abiotic Stress Responses in Plants. Int J Mol Sci 2510.3390/ijms251910548

Harris CJ, Amtmann A, Ton J (2023) Epigenetic processes in plant stress priming: Open questions and new approaches. Curr Opin Plant Biol 75:102432, https://doi.org/10.1016/j.pbi.2023.102432

Hereil A, Guillaume M, Duboscq R, Carretero Y, Pelpoir E, Bitton F, Giraud C, Karlova R, Testerink C, Stevens R, Causse M (2024) Characterisation of a major QTL for sodium accumulation in tomato grown in high salinity. Plant Cell Environ 47:5089-5103, https://doi.org/10.1111/pce.15082

Junaid MD, Chaudhry UK, Sanli BA, Gokce AF, Ozturk ZN (2024) A review of the potential involvement of small RNAs in transgenerational abiotic stress memory in plants. Funct Integr Genomics 24:74, https://doi.org/10.1007/s10142-024-01354-7

Kappel C, Friedrich T, Oberkofler V, Jiang L, Crawford T, Lenhard M, Baurle I (2023) Genomic and epigenomic determinants of heat stress-induced transcriptional memory in Arabidopsis. Genome Biol 24:129, https://doi.org/10.1186/s13059-023-02970-5

King J, Dreisigacker S, Reynolds M, Bandyopadhyay A, Braun HJ, Crespo-Herrera L, Crossa J, Govindan V, Huerta J, Ibba MI, Robles-Zazueta CA, Saint Pierre C, Singh PK, Singh RP, Achary VMM, Bhavani S, Blasch G, Cheng S, Dempewolf H, Flavell RB, Gerard G, Grewal S, Griffiths S, Hawkesford M, He X, Hearne S, Hodson D, Howell P, Jalal Kamali MR, Karwat H, Kilian B, King IP, Kishii M, Kommerell VM, Lagudah E, Lan C, Montesinos-Lopez OA, Nicholson P, Perez-Rodriguez P, Pinto F, Pixley K, Rebetzke G, Rivera-Amado C, Sansaloni C, Schulthess U, Sharma S, Shewry P, Subbarao G, Tiwari TP, Trethowan R, Uauy C (2024) Wheat genetic resources have avoided disease pandemics, improved food security, and reduced environmental footprints: A review of historical impacts and future opportunities. Glob Chang Biol 30:e17440, https://doi.org/10.1111/gcb.17440

Li W, Boer MP, Joosen RVL, Zheng C, Percival-Alwyn L, Cockram J, Van Eeuwijk FA (2024) Modeling QTL-by-environment interactions for multi-parent populations. Front Plant Sci 15:1410851, https://doi.org/10.3389/fpls.2024.1410851

Lin X, Yin J, Wang Y, Yao J, Li QQ, Latzel V, Bossdorf O, Zhang YY (2024) Environment-induced heritable variations are common in Arabidopsis thaliana. Nat Commun 15:4615, https://doi.org/10.1038/s41467-024-49024-3

Mohanavel V, Muthu V, Kambale R, Palaniswamy R, Seeli P, Ayyenar B, Rajagopalan V, Manickam S, Rajasekaran R, Rahman H, Nallathambi J, Swaminathan M, Chellappan G, Vellingiri G, Muthurajan R (2024) Marker-assisted breeding accelerates the development of multiple-stress-tolerant rice genotypes adapted to wider environments. Front Plant Sci 15:1402368, https://doi.org/10.3389/fpls.2024.1402368

Napier JD, Heckman RW, Juenger TE (2023) Gene-by-environment interactions in plants: Molecular mechanisms, environmental drivers, and adaptive plasticity. Plant Cell 35:109-124, https://doi.org/10.1093/plcell/koac322

Roychowdhury R, Ullah N, Ozturk-Gokce ZN, Budak H (2024) Haplotype Mapping Coupled Speed Breeding in Globally Diverse Wheat Germplasm for Genomics-Assisted Breeding:265-272, https://doi.org/10.1007/978-3-031-38294-9_13

Sahito JH, Zhang H, Gishkori ZGN, Ma C, Wang Z, Ding D, Zhang X, Tang J (2024) Advancements and Prospects of Genome-Wide Association Studies (GWAS) in Maize. Int J Mol Sci 2510.3390/ijms25031918

Singh A, Verma AK, Kumar S, Bag SK, Roy S (2024) Genome-wide DNA methylation and their transgenerational pattern differ in Arabidopsis thaliana populations originated along the elevation of West Himalaya. BMC Plant Biol 24:936, https://doi.org/10.1186/s12870-024-05641-0

Sun L, Wen J, Peng H, Yao Y, Hu Z, Ni Z, Sun Q, Xin M (2022) The genetic and molecular basis for improving heat stress tolerance in wheat. aBIOTECH 3:25-39, https://doi.org/10.1007/s42994-021-00064-z

Wang T, Wang F, Deng S, Wang K, Feng D, Xu F, Guo W, Yu J, Wu Y, Wuriyanghan H, Li ST, Gu X, Le L, Pu L (2025) Single-cell transcriptomes reveal spatiotemporal heat stress response in maize roots. Nat Commun 16:177, https://doi.org/10.1038/s41467-024-55485-3

Wei H, Wang X, Zhang Z, Yang L, Zhang Q, Li Y, He H, Chen D, Zhang B, Zheng C, Leng Y, Cao X, Cui Y, Shi C, Liu Y, Lv Y, Ma J, He W, Liu X, Xu Q, Yuan Q, Yu X, Wang T, Qian H, Li X, Zhang B, Zhang H, Chen W, Guo M, Dai X, Wang Y, Zheng X, Guo L, Xie X, Qian Q, Shang L (2024) Uncovering key salt-tolerant regulators through a combined eQTL and GWAS analysis using the super pan-genome in rice. Natl Sci Rev 11:nwae043, https://doi.org/10.1093/nsr/nwae043

Yang Z, Cao Y, Shi Y, Qin F, Jiang C, Yang S (2023) Genetic and molecular exploration of maize environmental stress resilience: Toward sustainable agriculture. Mol Plant 16:1496-1517, https://doi.org/10.1016/j.molp.2023.07.005

Zhang M, Kong D, Wang H (2023) Genomic landscape of maize domestication and breeding improvement. Seed Biology 2:0-0, https://doi.org/10.48130/SeedBio-2023-0009

Zhu F, Jadhav SS, Tohge T, Salem MA, Lee JM, Giovannoni JJ, Cheng Y, Alseekh S, Fernie AR (2022) A comparative transcriptomics and eQTL approach identifies SlWD40 as a tomato fruit ripening regulator. Plant Physiol 190:250-266, https://doi.org/10.1093/plphys/kiac200
